# Large language model–based simplification of breast imaging reports: a prospective multicentre study with 2965 patient-based evaluations

**DOI:** 10.1186/s13244-026-02423-0

**Published:** 2026-11-02

**Authors:** Veronica Magni, Matilde Pavan, Maria Cosentino, Maurizio Cè, Andrea Cozzi, Maria Iodice, Alessandro Liguori, Marta Montesano, Sveva Mortellaro, Luca Nicosia, Mariassunta Roberta Pannarale, Filippo Pesapane, Giulia Quercioli, Giulia Querques, Gianpaolo Carrafiello, Sonia Santicchia, Serena Carriero

**Affiliations:** 1 Postgraduate School in Radiodiagnostics, Università degli Studi di Milano, Milan, Italy; 2 Radiology and Interventional Radiology, Fondazione IRCCS Ca’ Granda Ospedale Maggiore Policlinico, Milan, Italy; 3 Institute of Integrated Diagnostics of Southern Switzerland (IDISI), Ente Ospedaliero Cantonale (EOC), Lugano, Switzerland; 4 Department of Biomedical, Surgical and Dental Sciences, Università degli Studi di Milano, Milan, Italy; 5 Breast Imaging Division, Radiology Department, IEO European Institute of Oncology IRCCS, Milan, Italy; 6 UOSD Senologia Ospedaliera, Ospedale A. Perrino, Brindisi, Italy; 7 Radiology Department, Ospedale di Stato–Istituto per la Sicurezza Sociale, Cailungo, San Marino; 8 Department of Oncology and Haemato-Oncology, Università degli Studi di Milano, Milan, Italy

**Keywords:** Breast, Artificial intelligence, Large language models, Patient preference, Surveys and questionnaires

## Abstract

**Objective:**

To compare patient perceptions of radiologist-written and artificial intelligence (AI)-generated simplifications of breast imaging reports in terms of simplicity, comprehensibility, empathy, and overall preference.

**Materials and methods:**

In this prospective multicentre survey-based study, 10 anonymized breast imaging reports (5 ultrasound, 5 mammography; one BI-RADS category 1–5 per modality) were simplified by ChatGPT-4 within a 50-word limit. After assessment by a panel of 20 physicians, paired radiologist-written and AI-generated reports were evaluated by 314 patients (2965 evaluations) who rated simplicity, comprehensibility, and empathy on a 5-point Likert scale and indicated their preferred report. The origin of each report was concealed, and each pair was presented in randomized order. Univariable and multivariable mixed-effects logistic regression analyses were performed to identify factors associated with preferring AI-generated reports.

**Results:**

AI-generated reports were preferred in 63.3% (1876/2965) of evaluations. High simplicity and comprehensibility scores (4–5) were more frequently assigned to AI reports than to radiologist-written reports (70.0% vs 43.4% and 68.1% vs 40.4%, respectively). In multivariable analysis, higher comprehensibility was the strongest predictor in both directions: higher comprehensibility of AI-generated reports increased the odds of preferring AI (score 4: adjusted OR 1.87, 95% CI 1.08–3.23, *p* = 0.025), while higher comprehensibility of radiologist-written reports reduced them (score 4: adjusted OR 0.45, 95% CI 0.29–0.69, *p* < 0.001). Preference for AI-generated reports showed a non-significant trend toward increase with educational level.

**Conclusion:**

AI-generated breast imaging reports were preferred by most patients and perceived as clearer, supporting a complementary and supervised use of AI-generated summaries.

**Key Points:**

***Question*** Patients increasingly access their own radiology reports, yet standard radiologist-written reports are often difficult to understand, highlighting the need for patient-friendly alternatives.

***Findings*** In this study, AI-generated breast imaging reports were preferred by patients in 63.3% of evaluations, with comprehensibility emerging as the strongest predictor of preference.

***Critical relevance statement*:**

AI-generated breast imaging reports were preferred by most patients and received higher ratings for simplicity and comprehensibility than radiologist-written reports, supporting a supervised and complementary use of AI-generated summaries in clinical breast imaging.

**Graphical Abstract:**

**Figure:**
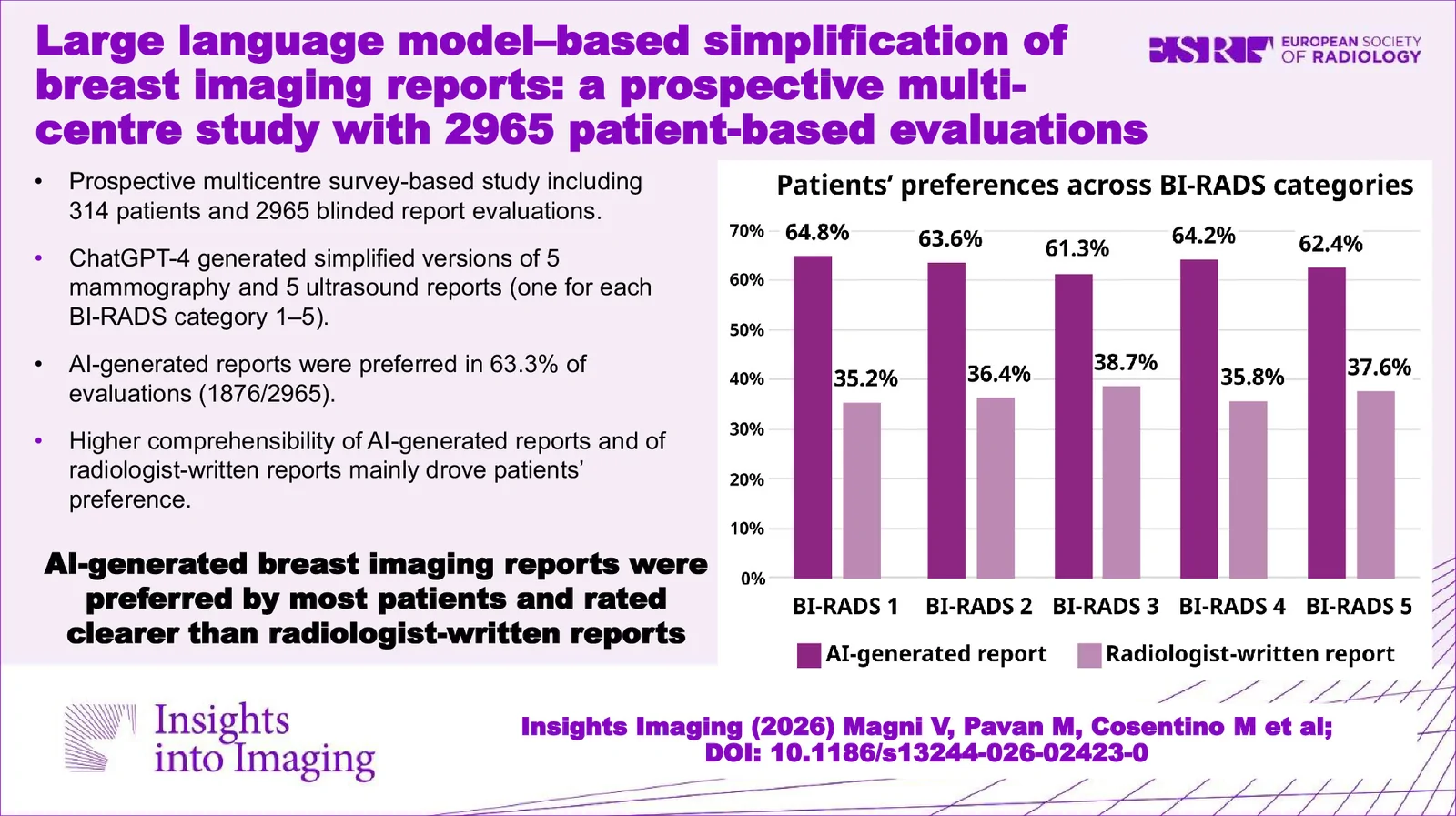

## Introduction

Historically, radiology reports have served as a means of communication primarily between radiologists and referring physicians [1]. Only in the late 20th century did radiologists begin to consider patients as direct recipients of their reports [2]. A transformative milestone in patient access to medical information was the enactment of the 21st Century Cures Act [3], which eliminated barriers to the sharing of health data and mandated near real-time access to electronic medical records [4]. This led to an increasing number of patients accessing their imaging results independently through electronic health record portals, which can facilitate more timely care and empower patients in the decision-making process [5, 6]. While this legislative framework is specific to the United States, other countries have developed analogous mechanisms for patient access to health records. In Italy—where this study was conducted—a national electronic health record (Fascicolo Sanitario Elettronico) was established in 2012 and reformed in 2023 [7, 8], providing patients with digital access to their clinical documents, including radiology reports [9], although patient engagement with the platform still sees marked regional heterogeneity.

Despite these advancements, several challenges persist. While patients consistently express a strong desire for digital access to their imaging results [10], their preferences vary when it comes to how those results are delivered—some favor direct access, while others prefer interpretation by a physician [11]. This ambivalence often stems from a difficulty in understanding radiology reports, which are frequently dense, highly technical, and filled with unfamiliar terminology [12, 13]. Significant discrepancies between the language used by radiologists and the comprehension level of patients contribute to this disconnection [14].

Studies show that radiology reports in the U.S. are written at a 13th-grade reading level, whereas the average American adult reads at an 8th-grade level [15]. Alarmingly, only 4.2% of radiology reports meet this basic readability threshold [16]. The impact of this communication gap is considerable: patients who do not understand their reports may experience confusion, distress, or increased anxiety—particularly when results are abnormal—and often seek further clarification through electronic messages or follow-up visits [1].

To address these challenges, several strategies have been explored. These include standardizing report formats and leveraging artificial intelligence (AI) solutions, particularly natural language processing, to generate simplified summaries of imaging findings [17]. Large language models (LLMs), such as ChatGPT-4, have shown promise in translating radiologic terminology into patient-friendly language [18]. In breast imaging, these tools have demonstrated the ability to produce simplified versions of reports, especially for Breast Imaging Reporting and Data System (BI-RADS) 1 and 2 categories, without omitting clinically relevant information [19, 20].

However, while prior work has primarily assessed technical performance, little is known about how patients actually perceive these AI-generated texts. This study aims to fill this gap by evaluating how patients assess the simplicity, comprehensibility, and empathy of mammography and breast ultrasound reports written by radiologists and AI. Moreover, it investigates how educational level influences these preferences, offering insights into the ability of LLMs to bridge the communication gap in radiology.

## Materials and methods

### Study design

This prospective multicentre study in the form of a survey was conducted between March 1st and July 31st, 2024, at the Breast Radiology Department of Fondazione IRCCS Ca’ Granda Ospedale Maggiore Policlinico (Center 1) and at the Breast Imaging Division of the European Institute of Oncology IRCCS (Center 2), both in Milan, Italy. In both Centers, promotional posters regarding the survey were displayed in the corridors and waiting areas of the breast radiology sections, informing that adult patients presenting to undergo breast cancer screening and breast diagnostic imaging could request the questionnaire from radiologists and radiographers of the staff and return it completed in a completely anonymous manner. Considering this full anonymity of the survey—which also implied voluntary consent from the participants—no specific approval was required according to the regulations of the Ethics Committee of the institution of the senior author (Fondazione IRCCS Ca’ Granda Ospedale Maggiore Policlinico, Milan, Italy).

### Report selection and AI-based simplification

Three breast radiologists (S.C., M.Co., and A.L.), each with at least 10 years of experience in breast imaging, retrospectively selected 10 representative breast imaging reports in total (5 ultrasound and 5 mammography) from the Radiology Information System of Center 1. The selected radiologist-written reports covered BI-RADS categories 1–5 and were anonymized before further processing. Each radiologist-written report was then simplified using an LLM (ChatGPT-4), prompted on February 1st, 2024, with the instruction: “*This is a medical report. Could you simplify it using 50 words?*” The 50-word limit was selected to ensure comparability across BI-RADS categories and imaging modalities, to address the patient need for concise, readable summaries, and to limit the overall burden of the questionnaire. For every original radiologist-written report, one corresponding AI-generated report was obtained, resulting in 10 matched pairs. All AI-generated reports were checked for obvious formatting or de-identification issues and then paired one-to-one with the corresponding radiologist-written reports for subsequent evaluation by participants.

### Survey development

Before evaluating patients’ perception of the paired breast imaging reports, 20 physicians (10 breast radiologists and 10 from other specializations including oncology, pathology and family medicine) completed a questionnaire (reported in the Supplementary Material, inclusive of all report pairs) to evaluate each pair of radiologist-written and AI-generated reports (BI-RADS 1–5 for mammography and ultrasound), being aware of the origin of each report. For every AI-generated report, physicians rated readability/simplicity, comprehensibility, and correctness on a 5-point Likert scale, indicated whether the report could be misleading; then they selected which version they personally preferred and which they considered more understandable for patients.

Subsequently, a structured questionnaire (reported in the Supplementary Material, inclusive of all report pairs) was developed to evaluate patients’ perception of the same paired reports. Each participant received one radiologist-written report and one AI-generated report, labeled as “Report A” and “Report B,” without any indication of which report was authored by a radiologist or generated by AI. For each pair, the order of presentation of the reports (radiologist-written first versus AI-generated first) was randomized, ensuring that participants remained completely unaware of the origin of individual reports. Participants rated each report using a 5-point Likert scale (1 = very low, 5 = very high) for three dimensions: simplicity, comprehensibility, and empathy. They were then asked to indicate which report they preferred. The questionnaire also collected demographic information, including gender, age range, and educational level.

### Statistical analysis

Descriptive statistics were used to summarize demographic characteristics and the distribution of Likert scores for simplicity, comprehensibility, and empathy. Categorical variables were reported as absolute frequencies and percentages. Likert-scale distributions were calculated based on the number of valid responses for each item, which varied slightly due to missing data. The *melogit* module in STATA (version MP 19; StataCorp) was used to fit univariable and multivariable mixed-effects binary logistic regression models (the latter considering only independent variables that showed a significant association with the dependent variable at an omnibus test after univariable regression), calculating unadjusted and adjusted odds ratios (ORs) and their 95% confidence intervals (CIs). These models evaluated the association between the preference for AI-generated reports (dependent variable, with the preference for radiologist-written reports as the reference value) and report ratings of simplicity, comprehensibility, empathy scores, and patients’ educational level (independent variables representing fixed effects), accounting for hierarchical data clustering according to institution, patient, and imaging modality (random effects). Missing values were infrequent (< 2%) and were handled using a complete-case approach for all analyses. Statistical significance for all analyses was set at *p* < 0.05.

## Results

### Physician evaluation of AI-generated reports

Twenty physicians provided 200 evaluations (100 mammography and 100 ultrasound reports) across BI-RADS categories 1–5. Ratings of AI-generated reports clustered in the most favorable response options (Table 1): for readability/simplicity, 169/200 evaluations (84.5%) described the reports as at least readable, for comprehensibility, 164/200 (82.0%) rated the reports at least understandable, and for correctness, 144/200 (72.0%) judged them at least correct. Only 21/200 evaluations (10.5%) considered the AI-generated reports potentially misleading. No major clinical errors, such as omission of laterality or misreporting of the BI-RADS category, were identified by physician reviewers across the evaluated report pairs. Although physicians more often preferred the radiologist-written reports (148/200, 74.0%), they judged the AI-generated reports as better suited for patients in most cases (162/200, 81.0%).

**Table 1:** Physicians’ evaluation of AI-generated breast imaging reports

		*n* (%)
Readability/simplicity (AI report)	Not readable/simple	0 (0.0%)
	Slightly readable/simple	4 (2.0%)
	Moderately readable/simple	27 (13.5%)
	Readable/simple	114 (57.0%)
	Highly readable/simple	55 (27.5%)
Comprehensibility (AI report)	Not understandable	0 (0.0%)
	Slightly understandable	2 (1.0%)
	Moderately understandable	34 (17.0%)
	Understandable	113 (56.5%)
	Highly understandable	51 (25.5%)
Correctness (AI report)	Not correct	0 (0.0%)
	Slightly correct	16 (8.0%)
	Moderately correct	40 (20.0%)
	Correct	114 (57.0%)
	Highly correct	30 (15.0%)
Potentially misleading (AI report)	Yes	21 (10.5%)
	No	179 (89.5%)
Preferred report by physicians	Radiologist-written	148 (74.0%)
	AI-generated	52 (26.0%)
Report judged better for patient	Radiologist-written	38 (19.0%)
	AI-generated	162 (81.0%)

### Patients’ preferences

A total of 314 participants completed the survey, corresponding to 2965 evaluations. As shown in Table 2, 194/314 (61.8%) of these participants were from Center 1, while 120/314 (38.2%) were from Center 2. Most evaluations were provided by female participants (299/314, 95.2%), while age distribution of the participants reflected the clinical setting of the two Centers, where both opportunistic and organized screening examinations are performed, with 174/314 participants (55.4%) being in the 45–75 age range, i.e., that which sees the implementation of organized screening in the region where the two centers are located. Participants had a wide range of educational backgrounds, with 208/314 (66.2%) participants having at least a high school diploma.

**Table 2:** Demographic data of the survey participants

		Total 314 participants	Center 1 194 participants	Center 2 120 participants
Sex	Female	299 (95.2%)	184 (94.8%)	115 (95.8%)
	Male	10 (3.2%)	5 (2.6%)	5 (4.2%)
	Non-binary	3 (1.0%)	3 (1.5%)	0 (0.0%)
	Prefer not to say	1 (0.3%)	1 (0.5%)	0 (0.0%)
	Unknown	1 (0.3%)	1 (0.5%)	0 (0.0%)
Age	< 20 years	6 (1.9%)	0 (0.0%)	6 (5.0%)
	20–25 years	15 (4.8%)	2 (1.0%)	13 (10.8%)
	25–30 years	25 (8.0%)	4 (2.1%)	21 (17.5%)
	30–35 years	26 (8.3%)	9 (4.6%)	17 (14.2%)
	35–40 years	13 (4.1%)	4 (2.1%)	9 (7.5%)
	40–45 years	43 (13.7%)	31 (16.0%)	12 (10.0%)
	45–50 years	31 (9.9%)	23 (11.9%)	8 (6.7%)
	50–55 years	31 (9.9%)	20 (10.3%)	11 (9.2%)
	55–60 years	43 (13.7%)	33 (17.0%)	10 (8.3%)
	60–65 years	18 (5.7%)	12 (6.2%)	6 (5.0%)
	65–70 years	30 (9.6%)	28 (14.4%)	2 (1.7%)
	70–75 years	21 (6.7%)	18 (9.3%)	3 (2.5%)
	75–80 years	5 (1.6%)	5 (2.6%)	0 (0.0%)
	> 80 years	7 (2.2%)	5 (2.6%)	2 (1.7%)
Educational level	Primary school	4 (1.3%)	0 (0.0%)	4 (3.3%)
	Middle school	53 (16.9%)	31 (16.0%)	22 (18.3%)
	Vocational school	47 (15.0%)	20 (10.3%)	27 (22.5%)
	High school	89 (28.3%)	51 (26.3%)	38 (31.7%)
	Bachelor’s degree	41 (13.1%)	20 (10.3%)	21 (17.5%)
	Master’s degree	50 (15.9%)	44 (22.7%)	6 (5.0%)
	Postgraduate studies	28 (8.9%)	26 (13.4%)	2 (1.7%)
	Unknown	2 (0.6%)	2 (1.0%)	0 (0.0%)

Among the 2965 responses, 1876 (63.3%) indicated a preference for AI-generated reports, while 1089 (36.7%) favored radiologist-written reports. Preferences between AI-generated and radiologist-written reports were consistently in favor of AI across all BI-RADS categories. In BI-RADS category 1, 390 evaluations (64.8%) favored AI-generated reports compared with 212 (35.2%) favoring radiologist-written reports, while in BI-RADS category 2, 384 (63.6%) favored AI-generated reports and 220 (36.4%) favored radiologist-written reports. In BI-RADS category 3, 363 evaluations (61.3%) preferred AI-generated reports and 229 (38.7%) preferred radiologist-written reports, and in BI-RADS category 4, 377 (64.2%) favored AI-generated reports compared with 210 (35.8%) favoring radiologist-written reports. A similar pattern was observed in BI-RADS category 5, where 362 evaluations (62.4%) preferred AI-generated reports and 218 (37.6%) preferred radiologist-written reports.

According to patients’ age, a preference for AI-generated reports was observed from the 30–35 age group onward, with AI preference ranging from 56.5% to 75.2% across all groups between 30 and 80 years (Table 3). The < 20 and 25–30 age groups were exceptions, showing a preference for radiologist-written reports (63.3% and 56.2%, respectively), while the 20–25 group showed only a marginal AI preference (80/149, 53.7%).

**Table 3:** Report preferences (AI-generated versus radiologist-written) stratified by patients’ age group

Age group	AI-generated, *n* (%)	Radiologist-written, *n* (%)	Total, *n*
< 20	22 (36.7%)	38 (63.3%)	60
20–25	80 (53.7%)	69 (46.3%)	149
25–30	109 (43.8%)	140 (56.2%)	249
30–35	147 (56.5%)	113 (43.5%)	260
35–40	80 (64.0%)	45 (36.0%)	125
40–45	316 (75.2%)	104 (24.8%)	420
45–50	199 (69.1%)	89 (30.9%)	288
50–55	179 (59.9%)	120 (40.1%)	299
55–60	233 (62.5%)	140 (37.5%)	373
60–65	82 (68.3%)	38 (31.7%)	120
65–70	185 (71.7%)	73 (28.3%)	258
70–75	115 (71.4%)	46 (28.6%)	161
75–80	35 (71.4%)	14 (28.6%)	49
> 80	94 (61.0%)	60 (39.0%)	154

### Perceived characteristics of reports

**Fig. 1:**
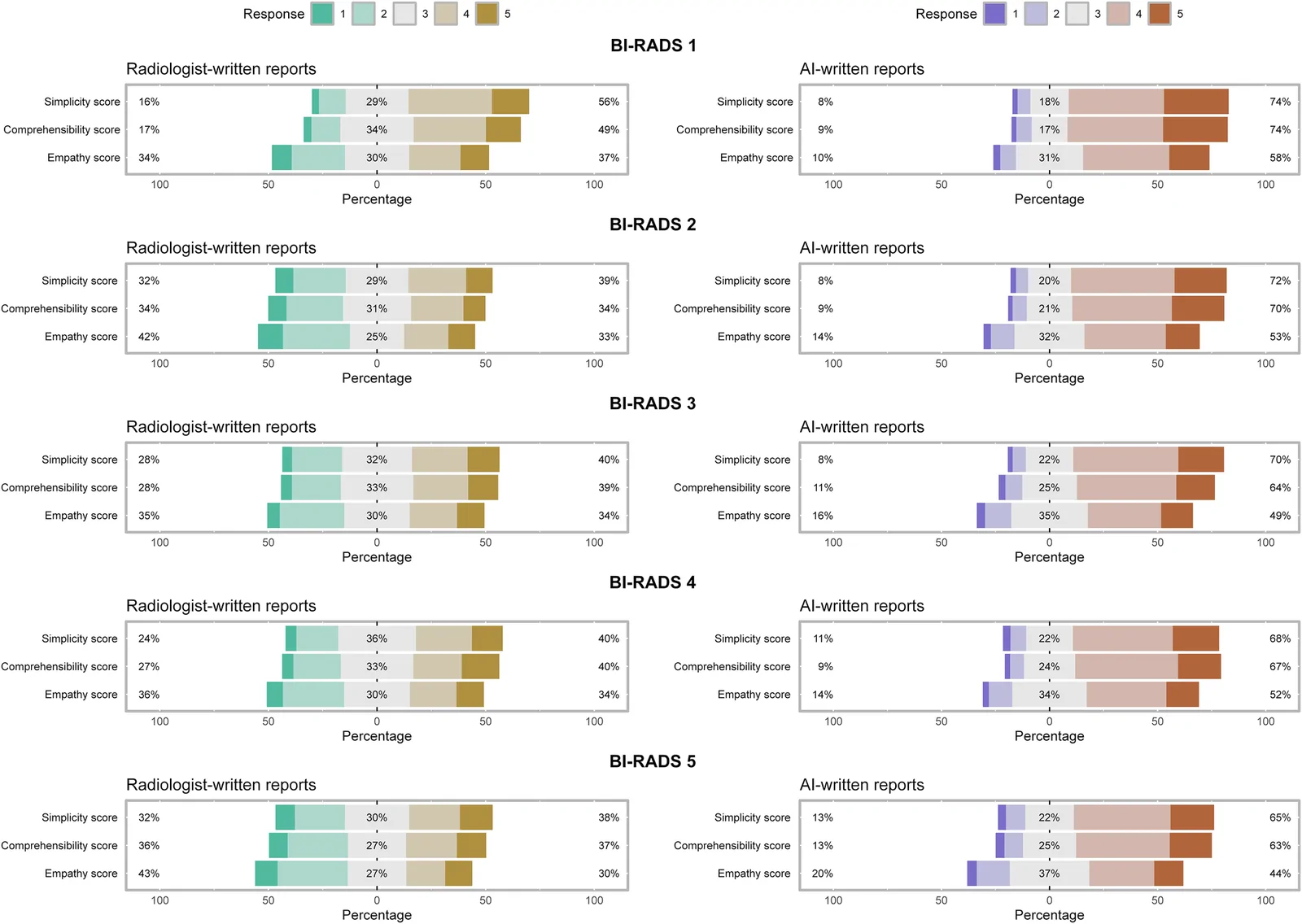
Patient ratings for simplicity, comprehensibility, and empathy of the reports according to each BI-RADS category, comparing radiologist-written reports and AI-generated reports. Ratings are expressed using a 5-point Likert scale and represented with divergent stacked barplots. The percentages represent, from left to right, the sum of low rating (1–2), medium rating (3), and high rating (4–5) categories, respectively

When evaluating the simplicity, comprehensibility, and empathy, participants systematically assigned higher scores to AI-generated reports than to radiologist-written reports (Table 4), a trend that was mirrored across BI-RADS categories (Fig. 1), particularly for lower ones (BI-RADS 1–2). Regression analyses included 2911 complete cases, with 54/2965 (1.8%) observations excluded due to missing values in Likert-scale variables.

**Table 4:** Ratings of simplicity, comprehensibility, and empathy for radiologist-written and AI-generated reports

	Radiologist-written report (%)	AI-generated report (%)
Simplicity score 1/5	178/2956 (6.0%)	84/2953 (2.8%)
Simplicity score 2/5	592/2956 (20.0%)	196/2953 (6.6%)
Simplicity score 3/5	906/2956 (30.7%)	608/2953 (20.6%)
Simplicity score 4/5	847/2956 (28.7%)	1375/2953 (46.6%)
Simplicity score 5/5	433/2956 (14.7%)	690/2953 (23.4%)
Comprehensibility score 1/5	181/2952 (6.1%)	81/2957 (2.7%)
Comprehensibility score 2/5	649/2952 (22.0%)	212/2957 (7.2%)
Comprehensibility score 3/5	930/2952 (31.5%)	652/2957 (22.1%)
Comprehensibility score 4/5	774/2952 (26.2%)	1350/2957 (45.7%)
Comprehensibility score 5/5	418/2952 (14.2%)	662/2957 (22.4%)
Empathy score 1/5	257/2926 (8.8%)	103/2928 (3.5%)
Empathy score 2/5	854/2926 (29.2%)	331/2928 (11.3%)
Empathy score 3/5	828/2926 (28.3%)	995/2928 (34.0%)
Empathy score 4/5	617/2926 (21.1%)	1042/2928 (35.6%)
Empathy score 5/5	370/2926 (12.6%)	457/2928 (15.6%)

In univariable regressions, all independent variables showed significant associations with the dependent variable at omnibus tests (*p* ≤ 0.029) and therefore entered multivariable regression. In both univariable and multivariable regressions, higher comprehensibility ratings for AI-generated reports were associated with high odds of preferring these reports (Table 5), with the most pronounced association observed at comprehensibility score 4 (unadjusted OR 1.69, 95% CI 1.00–2.83, *p* = 0.048; adjusted OR 1.87, 95% CI 1.08–3.23, *p* = 0.025). Empathy showed only a borderline trend with higher AI preference at higher scores, with the strongest effect at empathy score 4 (unadjusted OR 1.74, 95% CI 1.08–2.80, *p* = 0.022; adjusted OR 1.63, 95% CI 1.00–2.66, *p* = 0.051), whereas simplicity ratings did not influence patient preference.

**Table 5:** Univariable and multivariable mixed-effects binary logistic regression analysis for the preference of AI-generated reports

		Univariable regression		Multivariable regression	
		Unadjusted OR (95% CI)	*p*-value	Adjusted OR (95% CI)	*p*-value
Radiologist-written report	Simplicity score 1/5	Ref.	–	Ref.	–
	Simplicity score 2/5	1.33 (0.86–2.04)	0.198	1.14 (0.71–1.84)	0.583
	Simplicity score 3/5	0.76 (0.51–1.13)	0.178	0.78 (0.50–1.23)	0.288
	Simplicity score 4/5	0.44 (0.29–0.65)	< 0.001	0.56 (0.36–0.88)	0.012
	Simplicity score 5/5	0.49 (0.32–0.75)	0.001	0.54 (0.34–0.86)	0.010
	Comprehensibility score 1/5	Ref.	–	Ref.	–
	Comprehensibility score 2/5	1.20 (0.79–1.84)	0.389	0.98 (0.62–1.56)	0.936
	Comprehensibility score 3/5	0.74 (0.49–1.10)	0.136	0.77 (0.50–1.20)	0.256
	Comprehensibility score 4/5	0.37 (0.25–0.56)	< 0.001	0.45 (0.29–0.69)	< 0.001
	Comprehensibility score 5/5	0.58 (0.38–0.89)	0.012	0.60 (0.38–0.96)	0.033
	Empathy score 1/5	Ref.	–	Ref.	–
	Empathy score 2/5	1.01 (0.68–1.48)	0.965	1.15 (0.77–1.75)	0.488
	Empathy score 3/5	0.49 (0.34–0.71)	< 0.001	0.62 (0.42–0.93)	0.020
	Empathy score 4/5	0.40 (0.27–0.59)	< 0.001	0.58 (0.38–0.88)	0.010
	Empathy score 5/5	0.60 (0.40–0.91)	0.015	0.81 (0.52–1.25)	0.342
AI-generated report	Simplicity score 1/5	Ref.	–	Ref.	–
	Simplicity score 2/5	0.93 (0.52–1.65)	0.800	0.90 (0.50–1.63)	0.726
	Simplicity score 3/5	0.72 (0.43–1.21)	0.213	0.71 (0.41–1.21)	0.209
	Simplicity score 4/5	1.19 (0.72–1.98)	0.488	1.06 (0.62–1.79)	0.840
	Simplicity score 5/5	1.26 (0.75–2.12)	0.372	1.16 (0.68–1.97)	0.596
	Comprehensibility score 1/5	Ref.	–	Ref.	–
	Comprehensibility score 2/5	0.99 (0.55–1.76)	0.963	1.33 (0.73–2.41)	0.351
	Comprehensibility score 3/5	1.29 (0.77–2.19)	0.334	1.68 (0.97–2.92)	0.064
	Comprehensibility score 4/5	1.69 (1.00–2.83)	0.048	1.87 (1.08–3.23)	0.025
	Comprehensibility score 5/5	1.68 (0.99–2.84)	0.055	1.81 (1.04–3.13)	0.035
	Empathy score 1/5	Ref.	–	Ref.	–
	Empathy score 2/5	0.68 (0.41–1.15)	0.150	0.59 (0.35–1.00)	0.050
	Empathy score 3/5	1.08 (0.67–1.73)	0.757	0.98 (0.60–1.60)	0.940
	Empathy score 4/5	1.74 (1.08–2.80)	0.022	1.63 (1.00–2.66)	0.051
	Empathy score 5/5	1.15 (0.70–1.88)	0.579	1.06 (0.64–1.76)	0.812
Educational level	Primary school	Ref.	–	Ref.	–
	Middle school	1.17 (0.36–3.82)	0.793	1.02 (0.39–2.66)	0.965
	Vocational school	1.56 (0.35–3.76)	0.810	1.04 (0.40–2.70)	0.933
	High school	1.98 (0.62–6.34)	0.250	1.51 (0.59–3.88)	0.388
	Bachelor’s degree	1.88 (0.57–6.18)	0.301	1.52 (0.58–3.99)	0.391
	Master’s degree	2.19 (0.65–7.33)	0.204	1.57 (0.59–4.20)	0.367
	Postgraduate studies	2.02 (0.58–7.06)	0.271	1.72 (0.62–4.81)	0.299

*AI* artificial intelligence, *OR* odds ratio, *Ref.* reference

In contrast, higher simplicity, comprehensibility, and empathy ratings for radiologist-written reports were associated with decreased odds of preferring AI-generated reports, both at univariable and at multivariable regression analyses. Specifically, for simplicity, the strongest association with lower AI preference was observed at simplicity score 5, with an unadjusted OR of 0.49 (95% CI 0.32–0.75, *p* = 0.001) and an adjusted OR of 0.54 (95% CI 0.34–0.86, *p* = 0.010). For empathy, the most pronounced was seen at score 4, where the unadjusted OR was 0.40 (95% CI 0.27–0.59, *p* < 0.001) and the adjusted OR was 0.58 (95% CI 0.38–0.88, *p* = 0.010). Comprehensibility showed the strongest overall association with lower AI preference, particularly at score 4, with an unadjusted OR of 0.37 (95% CI 0.25–0.56, *p* < 0.001) and an adjusted OR of 0.45 (95% CI 0.29–0.69, *p* < 0.001).

Educational level showed a consistent trend toward higher preference of AI-generated reports with increasing education at univariable and multivariable analysis: in the latter, adjusted ORs ranged from 1.02 (95% CI 0.39–2.66) for middle school to 1.72 (95% CI 0.62–4.81) for postgraduate studies, but none of these associations reached statistical significance.

## Discussion

Since the implementation of the 21st Century Cures Act and the resulting expansion of patient access to medical records, concerns about the clarity and readability of radiology reports have become increasingly prominent. Many patients struggle to understand radiologist-written reports and experience confusion or anxiety when interpreting their findings [4, 6, 10, 11]. In this setting, LLMs have been proposed as an option to generate simplified, patient-friendly summaries of radiology reports, potentially improving readability and patient engagement without systematically omitting clinically relevant information, particularly in breast imaging [20, 21].

This survey-based study showed a clear patient preference for AI-generated breast imaging reports, which were preliminarily deemed to be suitable to be presented to patients in a physician-based evaluation, as they were considered at least readable in 84.5% and at least comprehensible in 82.0% of physician-based evaluations, and no major clinical errors were identified, although physicians still preferred radiologist-written reports in 74.0% of cases. In the patient-based survey, the AI-generated reports were selected by the participants as the better reports in 63.3% of evaluations: compared with radiologist-written reports, AI-generated reports were consistently rated higher across all evaluated domains, particularly in terms of simplicity and comprehensibility, suggesting that patients place substantial value on clear and accessible language when interpreting breast imaging results [22–25]. High simplicity scores (4 and 5) were assigned to 70.0% of AI-generated reports, compared with 43.4% of radiologist-written reports. Similarly, high comprehensibility scores (4 and 5) were reported in 68.1% of AI-generated reports versus 40.4% for radiologist-written reports, indicating a substantially greater perceived clarity of AI-generated content. Differences were also observed for empathy, although to a lesser extent. High empathy scores (4 and 5) were assigned to 51.2% of AI-generated reports, compared with 33.7% of radiologist-written reports. Despite this numerical advantage for AI, multivariable analysis showed that empathy attributed to radiologist-written reports played a protective role against preference for AI. Radiologist empathy scores of 3–4 were associated with a 38–42% reduction in the odds of preferring AI, underscoring the continued relevance of the human component in patient–radiologist communication.

In univariable and multivariable logistic regression analyses, the direction of associations was highly consistent, and multivariable models substantially confirmed the patterns observed in univariable analyses. For AI-generated reports, comprehensibility remained the strongest predictor of preferring AI: at score 4, the adjusted OR was 1.87. Empathy of AI-generated reports showed a borderline association at score 4 (adjusted OR 1.63), while simplicity showed only a non-significant trend. For radiologist-written reports, higher scores were associated with lower odds of preferring AI-generated reports, with the strongest effects observed for comprehensibility score 4 (adjusted OR 0.45), followed by simplicity score 5 (adjusted OR 0.54), simplicity score 4 (adjusted OR 0.56), and empathy score 4 (adjusted OR 0.58).

Educational level showed a consistent but non-significant trend toward higher AI preference with increasing education level, with adjusted ORs ranging from 1.02 for middle school to 1.72 for postgraduate studies. This pattern may reflect greater familiarity with digital technologies among highly educated individuals or a higher ability to recognize and appreciate the benefits of textual simplification [26].

These findings may be contextualized within the value-based radiology paradigm, which defines the value of radiological services not only in terms of diagnostic accuracy but also in terms of the quality of communication with patients [27]. Within this framework, report characteristics (including clarity and patient accessibility) are explicitly identified as key components, as also pointed out by the European Society of Radiology [28, 29]. In breast imaging specifically, patients frequently struggle to understand the implications of BI-RADS categories, and poor comprehension of mammography results has been associated with failure to complete appropriate follow-up [30]; thus, AI-generated summaries improving patient accessibility to the reports would not only enhance the perceived value of radiological reports but also positively impact patient care.

While these findings highlight the potential of AI-generated reports to enhance patient understanding, they also underscore the importance of ensuring that simplification does not compromise clinical accuracy [31, 32]. LLMs can generate plausible but clinically inaccurate content, a phenomenon known as hallucination, which represents a particular concern in medical contexts; however, published studies on LLM-based medical summaries have reported low hallucination rates, with major errors reducible through prompt and workflow refinement [33, 34]. Moreover, previous work has also shown that AI-generated summaries can maintain high factual accuracy and completeness for simpler breast imaging cases, particularly BI-RADS 1 and 2, whereas more complex categories pose greater challenges and require careful oversight [20, 35]. In our study, these two concerns were addressed by having both breast radiologists and general practitioners review the AI-generated reports prior to patient exposure, with only 10.5% being ultimately considered potentially misleading. These results support the overall clinical acceptability of AI-generated summaries and suggest that, while radiologist oversight remains necessary at the current stage, future advances in model performance may progressively reduce the extent of validation required before releasing these summaries. Notably, although physicians more often preferred the radiologist-written reports for their own clinical use (in 74.0% of cases), they judged the AI-generated reports as better suited for patients in the majority of cases (81.0% of cases), underscoring that these summaries are intended as a patient-facing communication tool rather than a substitute for the formal radiology report.

It should also be noted that the prompt used for simplification was intentionally minimal, ensuring reproducibility and reflecting a conservative real-world use consistent with analogous published studies [20, 25, 36]; given that ChatGPT-4 has been shown to achieve effective simplification even with such straightforward instructions [36], more sophisticated prompt engineering would likely further improve the quality of AI-generated outputs, and should be explored in future studies.

This study has some limitations that must be acknowledged. Only one LLM, ChatGPT-4 in the version available on February 1st, 2024, was evaluated, and no comparison was made with other general-purpose or domain-specific models; results may not generalize to other models or to more recent versions of the same model, given the rapid pace of LLMs development. However, the proven increase in reasoning capabilities of further versions of this and other models is likely to reinforce the observations gathered from this study, i.e., a preference for AI-generated reports. Second, all reports and AI-generated summaries were in Italian, potentially limiting the applicability of these results to other languages and cultural contexts. Furthermore, this study evaluated patient preference and perceived comprehensibility rather than objective comprehension; whether AI-generated summaries improve actual understanding remains to be investigated in future studies. Additionally, the voluntary nature of participation may have introduced selection bias, preferentially recruiting patients with higher health literacy or digital confidence. Finally, only ten report pairs were used for patient evaluation, a pragmatic choice that may not fully reflect the diversity and complexity of real-world breast imaging reports.

In conclusion, this prospective multicentre study suggests that AI-generated reports in breast imaging are perceived by patients as clearer and more empathetic than corresponding radiologist-written reports and are preferred in the majority of evaluations. When integrated into clinical workflows under direct radiologist supervision and validation, AI-generated summaries may serve as valuable communication aids to be systematically reviewed by a qualified radiologist prior to release, particularly in settings where direct interaction between patients and radiologists is limited. Future research should extend these findings to other imaging modalities, languages, and AI platforms and should investigate the impact of AI-generated reports on objective measures of patient understanding, anxiety, and downstream clinical decision-making.

## Supplementary information

ELECTRONIC SUPPLEMENTARY MATERIALS

## Data Availability

All data supporting the findings of this study are available within the paper.

## Abbreviations

AI: Artificial intelligence
BI-RADS: Breast Imaging Reporting and Data System
CI: Confidence interval
LLM: Large language model
OR: Odds ratio
