# Supplementary material for: Large language model–based simplification of breast imaging reports: a prospective multicentre study with 2965 patient-based evaluations
Source: Insights Imaging. 2026 Nov 2;17:273. doi: 10.1186/s13244-026-02423-0 (PMC13728069; doi:10.1186/s13244-026-02423-0)
Supplement: Supplementary file 1 [file 13244_2026_2423_MOESM1_ESM.pdf]

Large language model–based simplification of breast imaging reports: a prospective multicentre study with 2965 patient-based evaluations

ELECTRONIC SUPPLEMENTARY MATERIALS

QUESTIONNAIRE FOR PHYSICIANS

Dear participant,

Thank you very much for your availability. This brief questionnaire aims to assess the effectiveness of ChatGPT in simplifying breast imaging reports (mammography and ultrasound). We are interested in understanding whether the system's processing maintains the required level of accuracy and whether the reformulation introduces any errors that could lead to misinterpretation of the report. All data collected through this questionnaire will be handled anonymously.

DEMOGRAPHIC QUESTIONS

Sex

- Female
- Male
- Non-binary
- Prefer not to say

How old are you?

- <20
- 20–25
- 25–30
- 30–35
- 35–40
- 40–45
- 45–50
- 50–55
- 55–60
- 65–70
- 70–75
- 75–80
- >80

Are you a resident?

- First-year resident
- Second-year resident
- Third-year resident
- Fourth-year resident
- Not a resident

What is your medical specialty?

- Radiology
- General surgery
- Pathology
- Radiotherapy
- Oncology
- General practice / family medicine
- Other: ______

How much experience do you have in senology?

- <1 year
- 1–5 years
- 5–10 years
- >10 years

MAMMOGRAPHY

Read the two reports and complete the questionnaire. The first report is written by a radiologist; the second has been processed by ChatGPT, which was asked to summarize it in about 50 simple words.

Report A.
Mammographic appearance essentially unchanged compared with the previous examination: moderately dense breasts with scattered areas of fibroglandular density, with no suspicious focal lesions, also at the complementary breast ultrasound. No suspicious microcalcifications. Regular clinical and imaging follow-up is recommended, and earlier evaluation in case of new symptoms. ACR: b. BI-RADS: 1.

Report B.
The mammogram is similar to the previous one, with no worrisome findings. The breasts are moderately dense with some areas of denser, normal fibroglandular tissue, but there are no suspicious areas or abnormal calcifications. Regular follow-up is recommended. The BI-RADS result is 1, indicating no abnormalities.

In your opinion, is the AI-generated report readable/simple?

- 1 – The report is not readable
- 2 – The report is slightly readable
- 3 – The report is moderately readable
- 4 – The report is readable
- 5 – The report is very readable

In your opinion, is the AI-generated report understandable?

- 1 – The report is not understandable
- 2 – The report is slightly understandable
- 3 – The report is moderately understandable
- 4 – The report is understandable
- 5 – The report is very understandable

In your opinion, is the AI-generated report correct?

- 1 – The report is not correct
- 2 – The report is slightly correct
- 3 – The report is moderately correct
- 4 – The report is correct
- 5 – The report is highly correct

In your opinion, could the AI-generated report be misunderstood by patients and therefore be harmful?

- The report could be misunderstood
- The report could not be misunderstood

Which report do you prefer?

- Radiologist-written report
- AI-generated report

Which report is easier for patients to understand?

- Radiologist-written report
- AI-generated report

Report A.
Diagnostic question: screening. History: no family history; 2 pregnancies with breastfeeding. Clinical: asymptomatic patient, no suspicious palpable nodules. Clinical examination within normal limits. First mammographic examination. No previous ultrasound available for comparison. The patient reports a previous bilateral ultrasound with negative oncologic findings. Mammography performed in CC and MLO projections with tomosynthesis and synthetic reconstruction. Heterogeneously dense breasts; within the limits of evaluation given the high density, which reduces diagnostic sensitivity, no suspicious focal parenchymal alterations, microcalcifications, or pathological distortions are seen. Sparse round calcifications are present bilaterally. Annual clinical and imaging (mammography and ultrasound) follow-up is recommended, and earlier assessment if symptoms change. BI-RADS: 2. ACR: C.

Report B.
First screening mammogram in a patient without a family history of related diseases, with two previous pregnancies and breastfeeding and no clinical signs of suspicious nodules. The examination did not reveal worrisome abnormalities or pathological calcifications, although the breasts are quite dense, which may limit the sensitivity of the examination. Some scattered but non-worrisome calcifications are present. Annual follow-up is recommended. The BI-RADS result is 2, indicating benign findings.

In your opinion, is the AI-generated report readable/simple?

- 1 – The report is not readable
- 2 – The report is slightly readable
- 3 – The report is moderately readable
- 4 – The report is readable
- 5 – The report is very readable

In your opinion, is the AI-generated report understandable?

- 1 – The report is not understandable
- 2 – The report is slightly understandable
- 3 – The report is moderately understandable
- 4 – The report is understandable
- 5 – The report is very understandable

In your opinion, is the AI-generated report correct?

- 1 – The report is not correct
- 2 – The report is slightly correct
- 3 – The report is moderately correct
- 4 – The report is correct
- 5 – The report is highly correct

In your opinion, could the AI-generated report be misunderstood by patients and therefore be harmful?

- The report could be misunderstood
- The report could not be misunderstood

Which report do you prefer?

- Radiologist-written report
- AI-generated report

Which report is easier for patients to understand?

- Radiologist-written report
- AI-generated report

Report A.
Diagnostic question: microcalcifications, inverted left nipple. History: no family history. Inspection and clinical examination: no suspicious palpable findings; inverted left nipple. Mammography performed in CC and MLO projections with tomosynthesis and synthetic reconstruction. No previous examinations available. Breasts with predominantly fibro-fatty tissue and scattered residual fibroglandular tissue. Bilaterally, no suspicious focal parenchymal alterations or pathological distortions are seen. On the left, rod-like heterogeneous calcifications with linear distribution towards the nipple are observed; they appear benign at first impression but warrant 6-month mammographic follow-up because no prior images are available. No pathologically enlarged axillary lymph nodes. A targeted left-sided breast ultrasound in 6 months is recommended. ACR B. BI-RADS: 3.

Report B.
Patient with left nipple inversion and linear microcalcifications extending towards the nipple, which appear benign but require 6-month follow-up for confirmation, as no previous examinations are available for comparison. The breasts have predominantly fibro-fatty composition, without suspicious alterations. No enlarged lymph nodes are seen. A left breast ultrasound in 6 months is recommended. BI-RADS 3 suggests that the calcifications are probably benign.

In your opinion, is the AI-generated report readable/simple?

- 1 – The report is not readable
- 2 – The report is slightly readable
- 3 – The report is moderately readable
- 4 – The report is readable
- 5 – The report is very readable

In your opinion, is the AI-generated report understandable?

- 1 – The report is not understandable
- 2 – The report is slightly understandable
- 3 – The report is moderately understandable
- 4 – The report is understandable
- 5 – The report is very understandable

In your opinion, is the AI-generated report correct?

- 1 – The report is not correct
- 2 – The report is slightly correct
- 3 – The report is moderately correct
- 4 – The report is correct
- 5 – The report is highly correct

In your opinion, could the AI-generated report be misunderstood by patients and therefore be harmful?

- The report could be misunderstood
- The report could not be misunderstood

Which report do you prefer?

- Radiologist-written report
- AI-generated report

Which report is easier for patients to understand?

- Radiologist-written report
- AI-generated report

Report A.
Second-level examination performed to complete a screening mammogram. Family history of breast cancer in the patient's mother at 78 years. Clinical inspection and examination: negative. Mammography completed with tomosynthesis, showing a faint, well-defined opacity in the upper inner quadrant of the right breast, corresponding on ultrasound to an 8-mm, multilobulated hypoechoic lesion with benign appearance but, as a first finding, prudently considered worthy of histological characterization, which is performed on the same day. No additional suspicious focal lesions or bilateral axillary lymphadenopathy are detected. ACR B.

Report B.
Second-level examination performed after a screening mammogram. Although there are no evident symptoms, the exam revealed a small 8-mm opacity in the upper inner quadrant of the right breast, which appears benign but requires further histological assessment for confirmation. No other suspicious findings or abnormal axillary lymph nodes are present. The BI-RADS result is 4a, indicating a low suspicion of malignancy that requires further investigation.

In your opinion, is the AI-generated report readable/simple?

- 1 – The report is not readable
- 2 – The report is slightly readable
- 3 – The report is moderately readable
- 4 – The report is readable
- 5 – The report is very readable

In your opinion, is the AI-generated report understandable?

- 1 – The report is not understandable
- 2 – The report is slightly understandable
- 3 – The report is moderately understandable
- 4 – The report is understandable
- 5 – The report is very understandable

In your opinion, is the AI-generated report correct?

- 1 – The report is not correct
- 2 – The report is slightly correct
- 3 – The report is moderately correct
- 4 – The report is correct
- 5 – The report is highly correct

In your opinion, could the AI-generated report be misunderstood by patients and therefore be harmful?

- The report could be misunderstood
- The report could not be misunderstood

Which report do you prefer?

- Radiologist-written report
- AI-generated report

Which report is easier for patients to understand?

- Radiologist-written report
- AI-generated report

Report A.
Second-level examination performed to complete the mammographic assessment within the local screening programme. First-level mammography. Breast examination: at clinical evaluation, a large parenchymal density is palpable involving almost all outer quadrants. First-level mammography is completed with tomosynthesis. On the right, multiple parenchymal densities with architectural distortion are seen in all outer quadrants. On targeted ultrasound, three areas of pseudonodular echotexture alteration corresponding to these findings are identified:

- lower inner quadrant, maximum size about 10 × 9 mm;
- lower inner-outer quadrants, extended area about 40 × 16 mm;
- upper outer quadrant, additional focus about 13 × 12 mm.

In the ipsilateral axilla, a lymph node with blurred margins and thickened cortex, measuring about 8 × 8 mm, is seen. The described findings show overall suspicious mammographic and ultrasound features and require histological characterization by ultrasound-guided biopsy. On the left, no clearly suspicious parenchymal lesions are detected. In the central / lower inner region, a small area of relatively increased density with pseudo-distortive appearance is seen, already present on previous mammograms and without a clear ultrasound correlate. This finding is compatible with fibro-dysplastic changes but, prudently, is considered worthy of further evaluation with contrast-enhanced imaging. ACR C. BI-RADS 5 right. BI-RADS 3 left.

Report B.
Second-level examination performed after a screening mammogram. On the right breast, multiple dense, distorted areas are seen in the outer quadrants, with three pseudonodular areas ranging from 10 × 9 mm to 40 × 16 mm, and an axillary lymph node. These findings are considered suspicious and require further assessment with biopsy. On the left breast, there are no major alterations, only a denser area already seen on previous examinations, considered fibro-dysplastic in origin but requiring further evaluation with contrast-enhanced imaging. The BI-RADS results are 5 on the right, indicating high suspicion of malignancy, and 3 on the left, indicating probably benign findings.

In your opinion, is the AI-generated report readable/simple?

- 1 – The report is not readable
- 2 – The report is slightly readable
- 3 – The report is moderately readable
- 4 – The report is readable
- 5 – The report is very readable

In your opinion, is the AI-generated report understandable?

- 1 – The report is not understandable
- 2 – The report is slightly understandable
- 3 – The report is moderately understandable
- 4 – The report is understandable
- 5 – The report is very understandable

In your opinion, is the AI-generated report correct?

- 1 – The report is not correct
- 2 – The report is slightly correct
- 3 – The report is moderately correct
- 4 – The report is correct
- 5 – The report is highly correct

In your opinion, could the AI-generated report be misunderstood by patients and therefore be harmful?

- The report could be misunderstood
- The report could not be misunderstood

Which report do you prefer?

- Radiologist-written report
- AI-generated report

Which report is easier for patients to understand?

- Radiologist-written report
- AI-generated report

BREAST ULTRASOUND

Report A
Diagnostic question: breast ultrasound. Comparison with previous ultrasound. Asymptomatic patient. Family history of breast cancer (paternal aunt). The breast glands show a mixed echotexture. Bilaterally, no suspicious lesions are detected. No lymphadenopathy is seen in the axillary fossae. Annual ultrasound follow-up. BI-RADS 1.

Report B
The patient has no symptoms. Despite a family history of breast cancer (paternal aunt), no abnormalities or suspicious axillary lymph nodes are detected. The breast glands show a mixed echotexture. An annual ultrasound follow-up is recommended. The BI-RADS result is 1, indicating no abnormalities.

In your opinion, is the AI-generated report readable/simple?

- 1 – The report is not readable
- 2 – The report is slightly readable
- 3 – The report is moderately readable
- 4 – The report is readable
- 5 – The report is very readable

In your opinion, is the AI-generated report understandable?

- 1 – The report is not understandable
- 2 – The report is slightly understandable
- 3 – The report is moderately understandable
- 4 – The report is understandable
- 5 – The report is very understandable

In your opinion, is the AI-generated report correct?

- 1 – The report is not correct
- 2 – The report is slightly correct
- 3 – The report is moderately correct
- 4 – The report is correct
- 5 – The report is highly correct

In your opinion, could the AI-generated report be misunderstood by patients and therefore be harmful?

- The report could be misunderstood
- The report could not be misunderstood

Which report do you prefer?

- Radiologist-written report
- AI-generated report

Which report is easier for patients to understand?

- Radiologist-written report
- AI-generated report

Report A
Previous mammogram with BI-RADS 2. Asymptomatic patient. No family history of breast cancer. The breast glands show a mixed echotexture. Bilaterally, no suspicious lesions are detected. Multiple small anechoic formations consistent with cysts are present on both sides. Reactive-appearing lymph nodes are seen in the axillary fossae. Annual combined mammography and ultrasound follow-up is recommended. BI-RADS 2.

Report B
The patient, who has no symptoms and no family history of breast cancer, shows a mixed echotexture of the breast glands on ultrasound. No suspicious abnormalities are detected. Several small cysts are seen in both breasts, and the axillary lymph nodes appear reactive but not worrisome. An annual follow-up with mammography and ultrasound is recommended. The BI-RADS result is 2, indicating benign findings.

In your opinion, is the AI-generated report readable/simple?

- 1 – The report is not readable
- 2 – The report is slightly readable
- 3 – The report is moderately readable
- 4 – The report is readable
- 5 – The report is very readable

In your opinion, is the AI-generated report understandable?

- 1 – The report is not understandable
- 2 – The report is slightly understandable
- 3 – The report is moderately understandable
- 4 – The report is understandable
- 5 – The report is very understandable

In your opinion, is the AI-generated report correct?

- 1 – The report is not correct
- 2 – The report is slightly correct
- 3 – The report is moderately correct
- 4 – The report is correct
- 5 – The report is highly correct

In your opinion, could the AI-generated report be misunderstood by patients and therefore be harmful?

- The report could be misunderstood

- The report could not be misunderstood

Which report do you prefer?

- Radiologist-written report
- AI-generated report

Which report is easier for patients to understand?

- Radiologist-written report
- AI-generated report

Report A
Diagnostic question: fibrocystic breast disease. History: no family history. Previous ultrasound: 14-mm nodule in the lower outer quadrant of the right breast. The patient reports a mammogram with negative oncologic findings. Inspection and clinical examination: no suspicious palpable findings. Breasts with predominantly fibroglandular echotexture, without clearly suspicious focal lesions. Bilaterally, several small hypo-anechoic formations consistent with cysts. In the upper outer para-areolar region of the right breast, an oval, regular-walled hypoechoic lesion measuring 12 mm is seen, compatible with a fibroadenoma; given the first detection, a 6-month follow-up ultrasound is recommended, and a referral is provided to the patient. At the site of the previously reported lesion in the lower outer quadrant of the right breast, a small 4-mm hypo-anechoic area remains. No pathologically enlarged axillary lymph nodes. A 6-month ultrasound follow-up is recommended for the newly detected benign-appearing nodule in the upper outer right breast. BI-RADS 3.
Report B
Patient with a history of fibrocystic breast disease and no suspicious palpable findings. A recent mammogram was negative for cancer. Ultrasound shows small cystic lesions and a 12-mm nodule in the right breast, most likely a fibroadenoma. A follow-up ultrasound in 6 months is recommended. BI-RADS 3 suggests that the findings are probably benign.

In your opinion, is the AI-generated report readable/simple?

- 1 – The report is not readable
- 2 – The report is slightly readable
- 3 – The report is moderately readable
- 4 – The report is readable
- 5 – The report is very readable

In your opinion, is the AI-generated report understandable?

- 1 – The report is not understandable
- 2 – The report is slightly understandable
- 3 – The report is moderately understandable
- 4 – The report is understandable
- 5 – The report is very understandable

In your opinion, is the AI-generated report correct?

- 1 – The report is not correct
- 2 – The report is slightly correct
- 3 – The report is moderately correct
- 4 – The report is correct
- 5 – The report is highly correct

In your opinion, could the AI-generated report be misunderstood by patients and therefore be harmful?

- The report could be misunderstood
- The report could not be misunderstood

Which report do you prefer?

- Radiologist-written report
- AI-generated report

Which report is easier for patients to understand?

- Radiologist-written report
- AI-generated report

Report A
Ultrasound of the left chest wall after mastectomy shows a heterogeneous hypo-isoechoic nodular lesion of about 1 cm within the pre-pectoral fat tissue, presumably corresponding to the PET finding (differential diagnosis between fat necrosis and recurrence). A similar, smaller lesion is seen in the ipsilateral axilla. A cautious core needle biopsy is recommended for histological characterization, which is scheduled also in view of the language barrier (the presence of an accompanying person is recommended; alternatively, an interpreter will be arranged). BI-RADS 4a.

Report B
The results of a PET/CT and ultrasound of the left chest wall, where the patient previously underwent mastectomy, are reviewed. A roughly 1-cm, non-uniform nodular lesion is found in the pre-pectoral fat, which may represent fat necrosis or tumour recurrence. A similar but smaller lesion is also identified in the axilla. A biopsy is recommended to clarify the nature of these lesions. It is suggested that the patient be accompanied by an interpreter during the procedure. The BI-RADS result is 4a, indicating a suspicious abnormality.

In your opinion, is the AI-generated report readable/simple?

- 1 – The report is not readable
- 2 – The report is slightly readable
- 3 – The report is moderately readable
- 4 – The report is readable
- 5 – The report is very readable

In your opinion, is the AI-generated report understandable?

- 1 – The report is not understandable
- 2 – The report is slightly understandable
- 3 – The report is moderately understandable
- 4 – The report is understandable
- 5 – The report is very understandable

In your opinion, is the AI-generated report correct?

- 1 – The report is not correct
- 2 – The report is slightly correct
- 3 – The report is moderately correct
- 4 – The report is correct
- 5 – The report is highly correct

In your opinion, could the AI-generated report be misunderstood by patients and therefore be harmful?

- The report could be misunderstood
- The report could not be misunderstood

Which report do you prefer?

- Radiologist-written report
- AI-generated report

Which report is easier for patients to understand?

- Radiologist-written report
- AI-generated report

Report A
Diagnostic question: right axillary lymph nodes reported as suspicious on previous CT. Previous mammography and ultrasound in view. History of quadrantectomy in the upper outer quadrant of the right breast. The breast glands show a mixed echotexture. In the upper outer quadrant of the right breast, at the site of a previously described intramammary nodule on CT, a 5-mm avascular stellate lesion is seen at the lateral margin of the surgical scar; this lesion is suspicious and is sampled with an immediate core biopsy. In the lower inner quadrant of the right breast, a 2-mm round hypoechoic nodule is seen, with imaging features suggestive of benign fibro-dysplastic change. In the ipsilateral axilla, multiple lymphadenopathies (n>3) are present, the largest measuring 17 mm in maximum diameter with an 8-mm thick cortex; a core biopsy is performed on a 9-mm lymph node. BI-RADS 5.

Report B
Patient in follow-up after quadrantectomy of the right breast. A recent CT scan reported suspicious right axillary lymph nodes. Ultrasound identifies a 5-mm stellate lesion in the lateral portion of the surgical scar in the upper outer quadrant, which is biopsied because of suspicion of malignancy. A further 2-mm nodule, probably benign, is detected in the lower inner quadrant of the right breast. Multiple enlarged lymph nodes

are seen in the right axilla, and one of them is biopsied. The BI-RADS 5 category indicates a high suspicion of malignancy.

In your opinion, is the AI-generated report readable/simple?

- 1 – The report is not readable
- 2 – The report is slightly readable
- 3 – The report is moderately readable
- 4 – The report is readable
- 5 – The report is very readable

In your opinion, is the AI-generated report understandable?

- 1 – The report is not understandable
- 2 – The report is slightly understandable
- 3 – The report is moderately understandable
- 4 – The report is understandable
- 5 – The report is very understandable

In your opinion, is the AI-generated report correct?

- 1 – The report is not correct
- 2 – The report is slightly correct
- 3 – The report is moderately correct
- 4 – The report is correct
- 5 – The report is highly correct

In your opinion, could the AI-generated report be misunderstood by patients and therefore be harmful?

- The report could be misunderstood
- The report could not be misunderstood

Which report do you prefer?

- Radiologist-written report
- AI-generated report

Which report is easier for patients to understand?

- Radiologist-written report
- AI-generated report

QUESTIONNAIRE FOR PATIENTS

Dear participant,

Thank you very much for your willingness to take part in this survey-based study. This brief questionnaire is designed to assess the effectiveness of an artificial intelligence (AI) software in simplifying mammography and breast ultrasound reports.

You will read the radiologist's report and the report written by AI, without knowing which one was written by the radiologist and which one was generated by AI. After each pair of reports, you will be asked to express your preference by answering a few questions.

We are interested in assessing whether the text produced by AI is easier to understand than the radiologist's report.

All data collected through this questionnaire will be processed anonymously.

Thank you in advance for the time you will devote to completing this questionnaire.

DEMOGRAPHIC QUESTIONS

Sex

- Female
- Male
- Non-binary
- Prefer not to say

How old are you?

- <20
- 20–25
- 25–30
- 30–35
- 35–40
- 40–45
- 45–50
- 50–55
- 55–60
- 60–65
- 65–70
- 70–75
- 75–80
- >80

What is your highest level of education?

- Primary school certificate
- Middle school certificate
- Vocational school diploma
- High school diploma
- Bachelor’s degree
- Master’s degree
- Postgraduate degree

MAMMOGRAPHY

Report A
Mammographic appearance essentially unchanged compared with the previous examination: moderately dense breasts with scattered areas of fibroglandular density, with no suspicious focal lesions, also at the complementary breast ultrasound. No suspicious microcalcifications. Regular clinical and imaging follow-up is recommended, and earlier evaluation in case of new symptoms. ACR: b. BI-RADS: 1.

In your opinion, is this report readable/simple?

- 1 – The report is not readable
- 2 – The report is slightly readable
- 3 – The report is moderately readable
- 4 – The report is readable
- 5 – The report is very readable

In your opinion, is this report understandable?

- 1 – The report is not understandable
- 2 – The report is slightly understandable
- 3 – The report is moderately understandable
- 4 – The report is understandable
- 5 – The report is very understandable

In your opinion, is this report empathetic?

- 1 – The report is not empathetic at all
- 2 – The report is not empathetic
- 3 – The report is moderately empathetic
- 4 – The report is empathetic

- 5 – The report is very empathetic

In your opinion, is this report written by a physician or by an artificial intelligence system?

- 1 – Definitely by a physician
- 2 – Probably by a physician
- 3 – Could be written by either
- 4 – Probably written by an artificial intelligence system
- 5 – Definitely written by an artificial intelligence system

Report B
The mammogram is similar to the previous one, with no worrisome findings. The breasts are moderately dense with some areas of denser, normal fibroglandular tissue, but there are no suspicious areas or abnormal calcifications. Regular follow-up is recommended. The BI-RADS result is 1, indicating no abnormalities.

In your opinion, is this report readable/simple?

- 1 – The report is not readable
- 2 – The report is slightly readable
- 3 – The report is moderately readable
- 4 – The report is readable
- 5 – The report is very readable

In your opinion, is this report understandable?

- 1 – The report is not understandable
- 2 – The report is slightly understandable
- 3 – The report is moderately understandable
- 4 – The report is understandable
- 5 – The report is very understandable

In your opinion, is this report empathetic?

- 1 – The report is not empathetic at all
- 2 – The report is not empathetic
- 3 – The report is moderately empathetic
- 4 – The report is empathetic
- 5 – The report is very empathetic

In your opinion, is this report written by a physician or by an artificial intelligence system?

- 1 – Definitely by a physician
- 2 – Probably by a physician

- 3 – Could be written by either
- 4 – Probably written by an artificial intelligence system
- 5 – Definitely written by an artificial intelligence system

Which report do you think is better?

- Report A
- Report B

Report A
First screening mammogram in a patient without a family history of related diseases, with two previous pregnancies and breastfeeding and no clinical signs of suspicious nodules. The examination did not reveal worrisome abnormalities or pathological calcifications, although the breasts are quite dense, which may limit the sensitivity of the examination. Some scattered but non-worrisome calcifications are present. Annual follow-up is recommended. The BI-RADS result is 2, indicating benign findings.

In your opinion, is this report readable/simple?

- 1 – The report is not readable
- 2 – The report is slightly readable
- 3 – The report is moderately readable
- 4 – The report is readable
- 5 – The report is very readable

In your opinion, is this report understandable?

- 1 – The report is not understandable
- 2 – The report is slightly understandable
- 3 – The report is moderately understandable
- 4 – The report is understandable
- 5 – The report is very understandable

In your opinion, is this report empathetic?

- 1 – The report is not empathetic at all
- 2 – The report is not empathetic
- 3 – The report is moderately empathetic
- 4 – The report is empathetic
- 5 – The report is very empathetic

In your opinion, is this report written by a physician or by an artificial intelligence system?

- 1 – Definitely by a physician

- 2 – Probably by a physician
- 3 – Could be written by either
- 4 – Probably written by an artificial intelligence system
- 5 – Definitely written by an artificial intelligence system

Report B
Diagnostic question: screening. History: no family history; 2 pregnancies with breastfeeding. Clinical: asymptomatic patient, no suspicious palpable nodules. Clinical examination within normal limits. First mammographic examination. No previous ultrasound available for comparison. The patient reports a previous bilateral ultrasound with negative oncologic findings. Mammography performed in CC and MLO projections with tomosynthesis and synthetic reconstruction. Heterogeneously dense breasts; within the limits of evaluation given the high density, which reduces diagnostic sensitivity, no suspicious focal parenchymal alterations, microcalcifications, or pathological distortions are seen. Sparse round calcifications are present bilaterally. Annual clinical and imaging (mammography and ultrasound) follow-up is recommended, and earlier assessment if symptoms change. BI-RADS: 2. ACR: C.

In your opinion, is this report readable/simple?

- 1 – The report is not readable
- 2 – The report is slightly readable
- 3 – The report is moderately readable
- 4 – The report is readable
- 5 – The report is very readable

In your opinion, is this report understandable?

- 1 – The report is not understandable
- 2 – The report is slightly understandable
- 3 – The report is moderately understandable
- 4 – The report is understandable
- 5 – The report is very understandable

In your opinion, is this report empathetic?

- 1 – The report is not empathetic at all
- 2 – The report is not empathetic
- 3 – The report is moderately empathetic
- 4 – The report is empathetic
- 5 – The report is very empathetic

In your opinion, is this report written by a physician or by an artificial intelligence system?

- 1 – Definitely by a physician
- 2 – Probably by a physician

- 3 – Could be written by either
- 4 – Probably written by an artificial intelligence system
- 5 – Definitely written by an artificial intelligence system

Which report do you think is better?

- Report A
- Report B

Report A
Patient with left nipple inversion and linear microcalcifications extending towards the nipple, which appear benign but require 6-month follow-up for confirmation, as no previous examinations are available for comparison. The breasts have predominantly fibro-fatty composition, without suspicious alterations. No enlarged lymph nodes are seen. A left breast ultrasound in 6 months is recommended. BI-RADS 3 suggests that the calcifications are probably benign.

In your opinion, is this report readable/simple?

- 1 – The report is not readable
- 2 – The report is slightly readable
- 3 – The report is moderately readable
- 4 – The report is readable
- 5 – The report is very readable

In your opinion, is this report understandable?

- 1 – The report is not understandable
- 2 – The report is slightly understandable
- 3 – The report is moderately understandable
- 4 – The report is understandable
- 5 – The report is very understandable

In your opinion, is this report empathetic?

- 1 – The report is not empathetic at all
- 2 – The report is not empathetic
- 3 – The report is moderately empathetic
- 4 – The report is empathetic
- 5 – The report is very empathetic

In your opinion, is this report written by a physician or by an artificial intelligence system?

- 1 – Definitely by a physician

- 2 – Probably by a physician
- 3 – Could be written by either
- 4 – Probably written by an artificial intelligence system
- 5 – Definitely written by an artificial intelligence system

Report B
Diagnostic question: microcalcifications, inverted left nipple. History: no family history. Inspection and clinical examination: no suspicious palpable findings; inverted left nipple. Mammography performed in CC and MLO projections with tomosynthesis and synthetic reconstruction. No previous examinations available. Breasts with predominantly fibro-fatty tissue and scattered residual fibroglandular tissue. Bilaterally, no suspicious focal parenchymal alterations or pathological distortions are seen. On the left, rod-like heterogeneous calcifications with linear distribution towards the nipple are observed; they appear benign at first impression but warrant 6-month mammographic follow-up because no prior images are available. No pathologically enlarged axillary lymph nodes. A targeted left-sided breast ultrasound in 6 months is recommended. ACR B. BI-RADS: 3.

In your opinion, is this report readable/simple?

- 1 – The report is not readable
- 2 – The report is slightly readable
- 3 – The report is moderately readable
- 4 – The report is readable
- 5 – The report is very readable

In your opinion, is this report understandable?

- 1 – The report is not understandable
- 2 – The report is slightly understandable
- 3 – The report is moderately understandable
- 4 – The report is understandable
- 5 – The report is very understandable

In your opinion, is this report empathetic?

- 1 – The report is not empathetic at all
- 2 – The report is not empathetic
- 3 – The report is moderately empathetic
- 4 – The report is empathetic
- 5 – The report is very empathetic

In your opinion, is this report written by a physician or by an artificial intelligence system?

- 1 – Definitely by a physician
- 2 – Probably by a physician

- 3 – Could be written by either
- 4 – Probably written by an artificial intelligence system
- 5 – Definitely written by an artificial intelligence system

Which report do you think is better?

- Report A
- Report B

Report A
Second-level examination performed to complete a screening mammogram. Family history of breast cancer in the patient’s mother at 78 years. Clinical inspection and examination: negative. Mammography completed with tomosynthesis, showing a faint, well-defined opacity in the upper inner quadrant of the right breast, corresponding on ultrasound to an 8-mm, multilobulated hypoechoic lesion with benign appearance but, as a first finding, prudently considered worthy of histological characterization, which is performed on the same day. No additional suspicious focal lesions or bilateral axillary lymphadenopathy are detected. ACR B.

In your opinion, is this report readable/simple?

- 1 – The report is not readable
- 2 – The report is slightly readable
- 3 – The report is moderately readable
- 4 – The report is readable
- 5 – The report is very readable

In your opinion, is this report understandable?

- 1 – The report is not understandable
- 2 – The report is slightly understandable
- 3 – The report is moderately understandable
- 4 – The report is understandable
- 5 – The report is very understandable

In your opinion, is this report empathetic?

- 1 – The report is not empathetic at all
- 2 – The report is not empathetic
- 3 – The report is moderately empathetic
- 4 – The report is empathetic
- 5 – The report is very empathetic

In your opinion, is this report written by a physician or by an artificial intelligence system?

- 1 – Definitely by a physician

- 2 – Probably by a physician
- 3 – Could be written by either
- 4 – Probably written by an artificial intelligence system
- 5 – Definitely written by an artificial intelligence system

Report B
Second-level examination performed after a screening mammogram. Although there are no evident symptoms, the exam revealed a small 8-mm opacity in the upper inner quadrant of the right breast, which appears benign but requires further histological assessment for confirmation. No other suspicious findings or abnormal axillary lymph nodes are present. The BI-RADS result is 4a, indicating a low suspicion of malignancy that requires further investigation.

In your opinion, is this report readable/simple?

- 1 – The report is not readable
- 2 – The report is slightly readable
- 3 – The report is moderately readable
- 4 – The report is readable
- 5 – The report is very readable

In your opinion, is this report understandable?

- 1 – The report is not understandable
- 2 – The report is slightly understandable
- 3 – The report is moderately understandable
- 4 – The report is understandable
- 5 – The report is very understandable

In your opinion, is this report empathetic?

- 1 – The report is not empathetic at all
- 2 – The report is not empathetic
- 3 – The report is moderately empathetic
- 4 – The report is empathetic
- 5 – The report is very empathetic

In your opinion, is this report written by a physician or by an artificial intelligence system?

- 1 – Definitely by a physician
- 2 – Probably by a physician
- 3 – Could be written by either
- 4 – Probably written by an artificial intelligence system

- 5 – Definitely written by an artificial intelligence system

Which report do you think is better?

- Report A
- Report B

Report A
Second-level examination performed to complete the mammographic assessment within the local screening programme. First-level mammography. Breast examination: at clinical evaluation, a large parenchymal density is palpable involving almost all outer quadrants. First-level mammography is completed with tomosynthesis. On the right, multiple parenchymal densities with architectural distortion are seen in all outer quadrants. On targeted ultrasound, three areas of pseudonodular echotexture alteration corresponding to these findings are identified:

- lower inner quadrant, maximum size about 10 × 9 mm;
- lower inner-outer quadrants, extended area about 40 × 16 mm;
- upper outer quadrant, additional focus about 13 × 12 mm.

In the ipsilateral axilla, a lymph node with blurred margins and thickened cortex, measuring about 8 × 8 mm, is seen. The described findings show overall suspicious mammographic and ultrasound features and require histological characterization by ultrasound-guided biopsy. On the left, no clearly suspicious parenchymal lesions are detected. In the central / lower inner region, a small area of relatively increased density with pseudo-distortive appearance is seen, already present on previous mammograms and without a clear ultrasound correlate. This finding is compatible with fibro-dysplastic changes but, prudently, is considered worthy of further evaluation with contrast-enhanced imaging. ACR C. BI-RADS 5 right. BI-RADS 3 left.

In your opinion, is this report readable/simple?

- 1 – The report is not readable
- 2 – The report is slightly readable
- 3 – The report is moderately readable
- 4 – The report is readable
- 5 – The report is very readable

In your opinion, is this report understandable?

- 1 – The report is not understandable
- 2 – The report is slightly understandable
- 3 – The report is moderately understandable
- 4 – The report is understandable
- 5 – The report is very understandable

In your opinion, is this report empathetic?

- 1 – The report is not empathetic at all

- 2 – The report is not empathetic
- 3 – The report is moderately empathetic
- 4 – The report is empathetic
- 5 – The report is very empathetic

In your opinion, is this report written by a physician or by an artificial intelligence system?

- 1 – Definitely by a physician
- 2 – Probably by a physician
- 3 – Could be written by either
- 4 – Probably written by an artificial intelligence system
- 5 – Definitely written by an artificial intelligence system

Report B
Second-level examination performed after a screening mammogram. On the right breast, multiple dense, distorted areas are seen in the outer quadrants, with three pseudonodular areas ranging from 10 × 9 mm to 40 × 16 mm, and an axillary lymph node. These findings are considered suspicious and require further assessment with biopsy. On the left breast, there are no major alterations, only a denser area already seen on previous examinations, considered fibro-dysplastic in origin but requiring further evaluation with contrast-enhanced imaging. The BI-RADS results are 5 on the right, indicating high suspicion of malignancy, and 3 on the left, indicating probably benign findings.

In your opinion, is this report readable/simple?

- 1 – The report is not readable
- 2 – The report is slightly readable
- 3 – The report is moderately readable
- 4 – The report is readable
- 5 – The report is very readable

In your opinion, is this report understandable?

- 1 – The report is not understandable
- 2 – The report is slightly understandable
- 3 – The report is moderately understandable
- 4 – The report is understandable
- 5 – The report is very understandable

In your opinion, is this report empathetic?

- 1 – The report is not empathetic at all
- 2 – The report is not empathetic
- 3 – The report is moderately empathetic

- 4 – The report is empathetic
- 5 – The report is very empathetic

In your opinion, is this report written by a physician or by an artificial intelligence system?

- 1 – Definitely by a physician
- 2 – Probably by a physician
- 3 – Could be written by either
- 4 – Probably written by an artificial intelligence system
- 5 – Definitely written by an artificial intelligence system

Which report do you think is better?

- Report A
- Report B

BREAST ULTRASOUND

Report A
The patient has no symptoms. Despite a family history of breast cancer (paternal aunt), no abnormalities or suspicious axillary lymph nodes are detected. The breast glands show a mixed echotexture. An annual ultrasound follow-up is recommended. The BI-RADS result is 1, indicating no abnormalities.

In your opinion, is this report readable/simple?

- 1 – The report is not readable
- 2 – The report is slightly readable
- 3 – The report is moderately readable
- 4 – The report is readable
- 5 – The report is very readable

In your opinion, is this report understandable?

- 1 – The report is not understandable
- 2 – The report is slightly understandable
- 3 – The report is moderately understandable
- 4 – The report is understandable
- 5 – The report is very understandable

In your opinion, is this report empathetic?

- 1 – The report is not empathetic at all
- 2 – The report is not empathetic
- 3 – The report is moderately empathetic
- 4 – The report is empathetic
- 5 – The report is very empathetic

In your opinion, is this report written by a physician or by an artificial intelligence system?

- 1 – Definitely by a physician
- 2 – Probably by a physician
- 3 – Could be written by either
- 4 – Probably written by an artificial intelligence system
- 5 – Definitely written by an artificial intelligence system

Report B
Diagnostic question: breast ultrasound. Comparison with previous ultrasound. Asymptomatic patient. Family history of breast cancer (paternal aunt). The breast glands show a mixed echotexture. Bilaterally, no suspicious lesions are detected. No lymphadenopathy is seen in the axillary fossae. Annual ultrasound follow-up. BI-RADS 1.

In your opinion, is this report readable/simple?

- 1 – The report is not readable
- 2 – The report is slightly readable
- 3 – The report is moderately readable
- 4 – The report is readable
- 5 – The report is very readable

In your opinion, is this report understandable?

- 1 – The report is not understandable
- 2 – The report is slightly understandable
- 3 – The report is moderately understandable
- 4 – The report is understandable
- 5 – The report is very understandable

In your opinion, is this report empathetic?

- 1 – The report is not empathetic at all
- 2 – The report is not empathetic
- 3 – The report is moderately empathetic
- 4 – The report is empathetic
- 5 – The report is very empathetic

In your opinion, is this report written by a physician or by an artificial intelligence system?

- 1 – Definitely by a physician
- 2 – Probably by a physician
- 3 – Could be written by either
- 4 – Probably written by an artificial intelligence system
- 5 – Definitely written by an artificial intelligence system

Which report do you think is better?

- Report A
- Report B

Report A
Previous mammogram with BI-RADS 2. Asymptomatic patient. No family history of breast cancer. The breast glands show a mixed echotexture. Bilaterally, no suspicious lesions are detected. Multiple small anechoic formations consistent with cysts are present on both sides. Reactive-appearing lymph nodes are

seen in the axillary fossae. Annual combined mammography and ultrasound follow-up is recommended. BI-RADS 2.

In your opinion, is this report readable/simple?

- 1 – The report is not readable
- 2 – The report is slightly readable
- 3 – The report is moderately readable
- 4 – The report is readable
- 5 – The report is very readable

In your opinion, is this report understandable?

- 1 – The report is not understandable
- 2 – The report is slightly understandable
- 3 – The report is moderately understandable
- 4 – The report is understandable
- 5 – The report is very understandable

In your opinion, is this report empathetic?

- 1 – The report is not empathetic at all
- 2 – The report is not empathetic
- 3 – The report is moderately empathetic
- 4 – The report is empathetic
- 5 – The report is very empathetic

In your opinion, is this report written by a physician or by an artificial intelligence system?

- 1 – Definitely by a physician
- 2 – Probably by a physician
- 3 – Could be written by either
- 4 – Probably written by an artificial intelligence system
- 5 – Definitely written by an artificial intelligence system

Report B
The patient, who has no symptoms and no family history of breast cancer, shows a mixed echotexture of the breast glands on ultrasound. No suspicious abnormalities are detected. Several small cysts are seen in both breasts, and the axillary lymph nodes appear reactive but not worrisome. An annual follow-up with mammography and ultrasound is recommended. The BI-RADS result is 2, indicating benign findings.

In your opinion, is this report readable/simple?

- 1 – The report is not readable

- 2 – The report is slightly readable
- 3 – The report is moderately readable
- 4 – The report is readable
- 5 – The report is very readable

In your opinion, is this report understandable?

- 1 – The report is not understandable
- 2 – The report is slightly understandable
- 3 – The report is moderately understandable
- 4 – The report is understandable
- 5 – The report is very understandable

In your opinion, is this report empathetic?

- 1 – The report is not empathetic at all
- 2 – The report is not empathetic
- 3 – The report is moderately empathetic
- 4 – The report is empathetic
- 5 – The report is very empathetic

In your opinion, is this report written by a physician or by an artificial intelligence system?

- 1 – Definitely by a physician
- 2 – Probably by a physician
- 3 – Could be written by either
- 4 – Probably written by an artificial intelligence system
- 5 – Definitely written by an artificial intelligence system

Which report do you think is better?

- Report A
- Report B

Report A
Patient with a history of fibrocystic breast disease and no suspicious palpable findings. A recent mammogram was negative for cancer. Ultrasound shows small cystic lesions and a 12-mm nodule in the right breast, most likely a fibroadenoma. A follow-up ultrasound in 6 months is recommended. BI-RADS 3 suggests that the findings are probably benign.

In your opinion, is this report readable/simple?

- 1 – The report is not readable

- 2 – The report is slightly readable
- 3 – The report is moderately readable
- 4 – The report is readable
- 5 – The report is very readable

In your opinion, is this report understandable?

- 1 – The report is not understandable
- 2 – The report is slightly understandable
- 3 – The report is moderately understandable
- 4 – The report is understandable
- 5 – The report is very understandable

In your opinion, is this report empathetic?

- 1 – The report is not empathetic at all
- 2 – The report is not empathetic
- 3 – The report is moderately empathetic
- 4 – The report is empathetic
- 5 – The report is very empathetic

In your opinion, is this report written by a physician or by an artificial intelligence system?

- 1 – Definitely by a physician
- 2 – Probably by a physician
- 3 – Could be written by either
- 4 – Probably written by an artificial intelligence system
- 5 – Definitely written by an artificial intelligence system

Report B

Diagnostic question: fibrocystic breast disease. History: no family history. Previous ultrasound: 14-mm nodule in the lower outer quadrant of the right breast. The patient reports a mammogram with negative oncologic findings. Inspection and clinical examination: no suspicious palpable findings. Breasts with predominantly fibroglandular echotexture, without clearly suspicious focal lesions. Bilaterally, several small hypo-anechoic formations consistent with cysts. In the upper outer para-areolar region of the right breast, an oval, regular-walled hypoechoic lesion measuring 12 mm is seen, compatible with a fibroadenoma; given the first detection, a 6-month follow-up ultrasound is recommended, and a referral is provided to the patient. At the site of the previously reported lesion in the lower outer quadrant of the right breast, a small 4-mm hypo-anechoic area remains. No pathologically enlarged axillary lymph nodes. A 6-month ultrasound follow-up is recommended for the newly detected benign-appearing nodule in the upper outer right breast. BI-RADS 3.

In your opinion, is this report readable/simple?

- 1 – The report is not readable

- 2 – The report is slightly readable
- 3 – The report is moderately readable
- 4 – The report is readable
- 5 – The report is very readable

In your opinion, is this report understandable?

- 1 – The report is not understandable
- 2 – The report is slightly understandable
- 3 – The report is moderately understandable
- 4 – The report is understandable
- 5 – The report is very understandable

In your opinion, is this report empathetic?

- 1 – The report is not empathetic at all
- 2 – The report is not empathetic
- 3 – The report is moderately empathetic
- 4 – The report is empathetic
- 5 – The report is very empathetic

In your opinion, is this report written by a physician or by an artificial intelligence system?

- 1 – Definitely by a physician
- 2 – Probably by a physician
- 3 – Could be written by either
- 4 – Probably written by an artificial intelligence system
- 5 – Definitely written by an artificial intelligence system

Which report do you think is better?

- Report A
- Report B

Report A
Ultrasound of the left chest wall after mastectomy shows a heterogeneous hypo-isoechoic nodular lesion of about 1 cm within the pre-pectoral fat tissue, presumably corresponding to the PET finding (differential diagnosis between fat necrosis and recurrence). A similar, smaller lesion is seen in the ipsilateral axilla. A cautious core needle biopsy is recommended for histological characterization, which is scheduled also in view of the language barrier (the presence of an accompanying person is recommended; alternatively, an interpreter will be arranged). BI-RADS 4a.

In your opinion, is this report readable/simple?

- 1 – The report is not readable
- 2 – The report is slightly readable
- 3 – The report is moderately readable
- 4 – The report is readable
- 5 – The report is very readable

In your opinion, is this report understandable?

- 1 – The report is not understandable
- 2 – The report is slightly understandable
- 3 – The report is moderately understandable
- 4 – The report is understandable
- 5 – The report is very understandable

In your opinion, is this report empathetic?

- 1 – The report is not empathetic at all
- 2 – The report is not empathetic
- 3 – The report is moderately empathetic
- 4 – The report is empathetic
- 5 – The report is very empathetic

In your opinion, is this report written by a physician or by an artificial intelligence system?

- 1 – Definitely by a physician
- 2 – Probably by a physician
- 3 – Could be written by either
- 4 – Probably written by an artificial intelligence system
- 5 – Definitely written by an artificial intelligence system

Report B
The results of a PET/CT and ultrasound of the left chest wall, where the patient previously underwent mastectomy, are reviewed. A roughly 1-cm, non-uniform nodular lesion is found in the pre-pectoral fat, which may represent fat necrosis or tumour recurrence. A similar but smaller lesion is also identified in the axilla. A biopsy is recommended to clarify the nature of these lesions. It is suggested that the patient be accompanied by an interpreter during the procedure. The BI-RADS result is 4a, indicating a suspicious abnormality.

In your opinion, is this report readable/simple?

- 1 – The report is not readable
- 2 – The report is slightly readable
- 3 – The report is moderately readable

- 4 – The report is readable
- 5 – The report is very readable

In your opinion, is this report understandable?

- 1 – The report is not understandable
- 2 – The report is slightly understandable
- 3 – The report is moderately understandable
- 4 – The report is understandable
- 5 – The report is very understandable

In your opinion, is this report empathetic?

- 1 – The report is not empathetic at all
- 2 – The report is not empathetic
- 3 – The report is moderately empathetic
- 4 – The report is empathetic
- 5 – The report is very empathetic

In your opinion, is this report written by a physician or by an artificial intelligence system?

- 1 – Definitely by a physician
- 2 – Probably by a physician
- 3 – Could be written by either
- 4 – Probably written by an artificial intelligence system
- 5 – Definitely written by an artificial intelligence system

Which report do you think is better?

- Report A
- Report B

Report A
Patient in follow-up after quadrantectomy of the right breast. A recent CT scan reported suspicious right axillary lymph nodes. Ultrasound identifies a 5-mm stellate lesion in the lateral portion of the surgical scar in the upper outer quadrant, which is biopsied because of suspicion of malignancy. A further 2-mm nodule, probably benign, is detected in the lower inner quadrant of the right breast. Multiple enlarged lymph nodes are seen in the right axilla, and one of them is biopsied. The BI-RADS 5 category indicates a high suspicion of malignancy.

In your opinion, is this report readable/simple?

- 1 – The report is not readable
- 2 – The report is slightly readable

- 3 – The report is moderately readable
- 4 – The report is readable
- 5 – The report is very readable

In your opinion, is this report understandable?

- 1 – The report is not understandable
- 2 – The report is slightly understandable
- 3 – The report is moderately understandable
- 4 – The report is understandable
- 5 – The report is very understandable

In your opinion, is this report empathetic?

- 1 – The report is not empathetic at all
- 2 – The report is not empathetic
- 3 – The report is moderately empathetic
- 4 – The report is empathetic
- 5 – The report is very empathetic

In your opinion, is this report written by a physician or by an artificial intelligence system?

- 1 – Definitely by a physician
- 2 – Probably by a physician
- 3 – Could be written by either
- 4 – Probably written by an artificial intelligence system
- 5 – Definitely written by an artificial intelligence system

Report B

Diagnostic question: right axillary lymph nodes reported as suspicious on previous CT. Previous mammography and ultrasound in view. History of quadrantectomy in the upper outer quadrant of the right breast. The breast glands show a mixed echotexture. In the upper outer quadrant of the right breast, at the site of a previously described intramammary nodule on CT, a 5-mm avascular stellate lesion is seen at the lateral margin of the surgical scar; this lesion is suspicious and is sampled with an immediate core biopsy. In the lower inner quadrant of the right breast, a 2-mm round hypoechoic nodule is seen, with imaging features suggestive of benign fibro-dysplastic change. In the ipsilateral axilla, multiple lymphadenopathies (n>3) are present, the largest measuring 17 mm in maximum diameter with an 8-mm thick cortex; a core biopsy is performed on a 9-mm lymph node. BI-RADS 5.

In your opinion, is this report readable/simple?

- 1 – The report is not readable
- 2 – The report is slightly readable
- 3 – The report is moderately readable

- 4 – The report is readable
- 5 – The report is very readable

In your opinion, is this report understandable?

- 1 – The report is not understandable
- 2 – The report is slightly understandable
- 3 – The report is moderately understandable
- 4 – The report is understandable
- 5 – The report is very understandable

In your opinion, is this report empathetic?

- 1 – The report is not empathetic at all
- 2 – The report is not empathetic
- 3 – The report is moderately empathetic
- 4 – The report is empathetic
- 5 – The report is very empathetic

In your opinion, is this report written by a physician or by an artificial intelligence system?

- 1 – Definitely by a physician
- 2 – Probably by a physician
- 3 – Could be written by either
- 4 – Probably written by an artificial intelligence system
- 5 – Definitely written by an artificial intelligence system

Which report do you think is better?

- Report A
- Report B
